# Supplementary material for: Stacking Model‐Based Classifiers for Dealing With Multiple Sets of Noisy Labels
Source: Biom J. 2025 Mar 12;67(2):e70042. doi: 10.1002/bimj.70042 (PMC11898607; doi:10.1002/bimj.70042)
Supplement: Supplementary file 1 — Supporting Information [file BIMJ-67-e70042-s001.zip › density-based-ensemble-model-main/competitors_real_data/netlab-master/netlab-master/help/netderiv.htm]

Netlab Reference Manual netderiv


# netderiv

## Purpose

Evaluate derivatives of network outputs by weights generically.

## Synopsis

```
g = netderiv(w, net, x)
```

## Description

`g = netderiv(w, net, x)` takes a weight vector `w` and a network
data structure `net`, together with the matrix `x` of input
vectors, and returns the
gradient of the outputs with respect to the weights evaluated at `w`.

## See Also

`netevfwd`, `netopt`

---

**Pages:**
Index


---

Copyright (c) Ian T Nabney (1996-9)
